# Impact of the COVID-19 Pandemic on Colorectal and Prostate Cancer Screening in a Large U.S. Health System

**DOI:** 10.3390/healthcare10020264

**Published:** 2022-01-29

**Authors:** Amar H. Kelkar, Jing Zhao, Shu Wang, Christopher R. Cogle

**Affiliations:** 1 Division of Hematology and Oncology, Department of Medicine, College of Medicine, University of Florida, Gainesville, FL 32610, USA; amarhkelkar@gmail.com; 2 Department of Biostatistics, College of Public Health and Health Professions, University of Florida, Gainesville, FL 32610, USA; jingzhao@iastate.edu (J.Z.); swang0221@ufl.edu (S.W.)

**Keywords:** cancer screening, colorectal cancer, prostate cancer, COVID-19, health disparities, health policy, public health

## Abstract

During the first year of the coronavirus disease 2019 (COVID-19) pandemic, prevention measures included quarantining and facility closures. Because cancer screening is dependent on interventions in facilities, the extent of the COVID-19 impact on screening was questioned. A claims registry from a large health system was queried for colorectal and prostate cancer screening. A screening gap and screening loss ratio were calculated by comparing 2020 screening to historical reference years. All cancer screenings decreased in the first four months of the pandemic. Colorectal cancer screening returned to baseline in the latter six months of 2020. Prostate cancer screening exceeded baseline in the latter six months, but with a lesser gain than previous years. Populations disproportionately affected by decreased cancer screening included men and black people. To catch-up after the initial deficit in screening, it is estimated that the rate of colorectal cancer screening needs to increase by 50%.

## 1. Introduction

Early in the coronavirus disease 2019 (COVID-19) pandemic, various disease models warned of excess cancer deaths due to missed cancer screenings resulting from a combination of quarantining the general population and a scarcity of health care resources [1,2]. Moreover, in some states, such as Florida, state governments prohibited non-essential health care services by executive orders.

In the early months of the COVID-19 pandemic, significant decreases in cancer screening were observed, with one study reporting reductions in screenings of breast cancer by 85%, colon cancer by 75%, prostate cancer by 74%, and lung cancer by 56% [3,4,5]. There has been speculation of tens of thousands of excess cancer deaths in the United States based on these screening calculations.

However, few studies have reported follow-up data on cancer screening after the initial waves of COVID-19. The general supposition is that if cancer screening rates do not supersede historical rates before the pandemic, then the population will suffer from a higher incidence of later-stage cancer diagnoses and possibly excess cancer deaths. However, if cancer screening rates supersede pre-pandemic levels, then a catch-up may be possible.

Another limitation in studying the pandemic’s impact on cancer screening has been the methods used for quantifying deficits. In this study, a new method called the “screening loss ratio” is introduced.

Herein, we present the findings of a retrospective database study that aimed to quantify the impact of the COVID-19 pandemic on colorectal and prostate cancer screenings in 2020 by comparing them to pre-pandemic reference screening levels. The secondary aims included measuring the impacts on screening for sub-populations.

## 2. Materials and Methods

### 2.1. Patient Data

This study was a single-center, retrospective database examination of patients who underwent screening for colorectal cancer or prostate cancer from 1 January 2017 to 31 December 2020 in the University of Florida (UF) Health system. Colorectal cancer screening was chosen as representative of a more invasive, longer-interval screening intervention in contrast to prostate cancer screening, which is less invasive with shorter time intervals. Colorectal and prostate cancer screenings were also chosen for examination given the established guidelines from the United States Preventive Services Task Force (USPSTF) and standards defined by the United States Centers for Disease Control (CDC). Procedures that meet the criteria for colon cancer screening in patients aged 50 to 75 include annual guaiac-based fecal occult blood test (gFOBT), annual fecal immunochemical test (FIT), FIT-DNA testing every 3 years, flexible sigmoidoscopy every 5 years, CT colonography every 5 years, and colonoscopy every 10 years. Procedures that meet the criteria for prostate cancer screening in patients aged 55 to 69 include prostate specific antigen (PSA) testing and digital rectal examination (DRE).

This study was approved by the University of Florida Institutional Review Board (IRB-01). The study included all patients 18 years old or older who underwent colorectal cancer or prostate cancer screenings during the study period. In general, clinicians used USPSTF guidelines for performing cancer screenings. An integrated data repository (IDR) at UF Health containing billing data was queried in January 2021 using diagnostic and procedure billing codes for colorectal cancer screening or prostate cancer screening (Supplementary Materials). Billing codes for colorectal cancer screening included CPT codes 45378, 82270, 82274, HCPCS codes G0105, G0121, G0328, or ICD-10 code Z12.11. Billing codes for prostate cancer screening included HCPCS codes G0102, G0103, or ICD-10 code Z12.5. For each study patient and particular cancer screening (colorectal or prostate), the first cancer screening per calendar year was used as the incident screening event. If there were multiple claims per patient for colorectal cancer screening or prostate cancer screening within the same calendar year, then only the first claim was used as the incident claim for the respective screening that year.

The UF Health Shands Hospital and Clinics are located in Alachua County within the State of Florida, though the catchment area extends to 23 counties in Northern Florida including Alachua, Baker, Bradford, Citrus, Clay, Columbia, Dixie, Gadsden, Gilchrist, Hamilton, Jefferson, Lafayette, Lake, Leon, Levy, Madison, Marion, Putnam, Sumter, Suwannee, Taylor, Union, and Wakulla counties.

### 2.2. Statistical Analyses

All statistical analyses were performed using IBM SPSS Statistics (Armonk, NY, USA). Descriptive statistics such as frequencies, means, and medians were used to analyze patient characteristics and screening frequency.

Historical reference screening rates of colorectal cancer or prostate cancer screening were calculated using an average of screening encounters from 2017 to 2019. When evaluating the demographic characteristics of the patients, data were collected on sex (male, female, missing), race (American Indian, Asian, Black, Multiracial, Other, Pacific Islander, White, missing), and ethnicity (Hispanic, Non-Hispanic, missing). Given the small number of patients with American Indian, Asian, Hispanic, Multiracial, Other, or Pacific Islander backgrounds, these racial groups were categorized as “Other” for statistical analyses.

The deficit in screening between 2020 and the reference years was termed the “Gap” and was calculated by subtracting the number of incident screening events in 2020 from the average number of incident screening events in the reference years. In Section 3.2, the Gap was calculated specifically for the months of March, April, May, and June 2020 compared to the same time period in the reference years to analyze the acute effects of the pandemic during a time when healthcare facilities had significantly restricted operations and community quarantining was widespread. In Section 3.3, the Gap was also calculated for the entire 2020 calendar year compared to the yearly averages of the reference years to analyze the totality of the effects of the pandemic in 2020 as healthcare facilities initially closed then reopened in July 2020 and community quarantining practices ebbed. The Gap is represented by a positive number when the number of screening events decreased. The purpose of calculating the Gap was to account for absolute measures of change in screening.

The screening loss ratio (SLR) was calculated by dividing the Gap by the monthly average of the reference years. The purpose of calculating the SLR was to describe the relative measures of change in screening, which permitted a comparison among sub-populations. Positive SLR values indicate a decrease in screening, while negative SLR values indicate screening gains. The magnitude of the SLR value indicates the relative extent of the decrease or increase in screening.

## 3. Results

### 3.1. Cancer Screening in Reference Years

During the reference years (2017–2019), there were 98,497 colon cancer screening encounters, corresponding to 69,937 unique patients and 34,039 prostate cancer screening encounters, corresponding to 20,002 unique patients.

The demographics of patients undergoing colorectal cancer screening during the study period are shown in Table 1. Colorectal cancer screening was more frequently performed in women, white patients, and those of non-Hispanic ethnicity.

The demographics of patients undergoing prostate cancer screening during the study period are shown in Table 2. Prostate screening was more frequently performed in white patients and those of non-Hispanic ethnicity.

**Figure 1:**
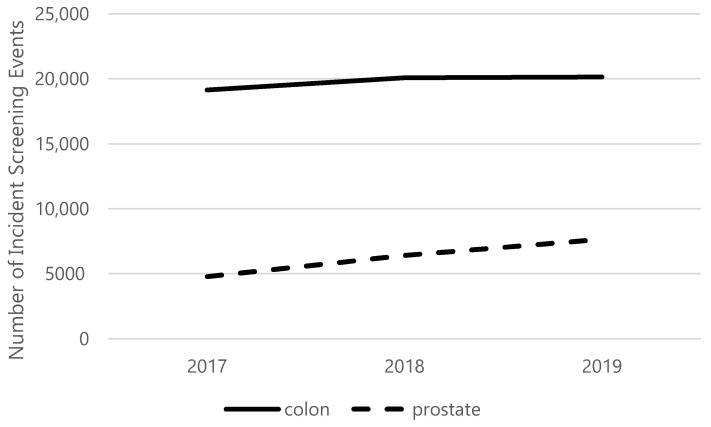
Incident of cancer screening in historical reference years 2017–2019.

Screening remained relatively steady during the reference years of 2017–2019 for colorectal cancer, but screening increased annually for prostate cancer during the reference years (Figure 1). The historical averages over the three reference years before 2020 were calculated to be 19,793 patients undergoing colorectal cancer screening and 6295 patients undergoing prostate cancer screening.

### 3.2. Cancer Screenings during March to June 2020

During the first four months of the COVID-19 pandemic in the US (March, April, May, June 2020), colorectal cancer screening decreased by 1891 patients compared to the same months in the reference years (Table 3). However, prostate cancer screening increased by 279 patients compared to the same four-month period in previous years (Table 4). It is noted that in the reference period, prostate cancer screening increased by 33% from 2017 to 2018 and then by 19% from 2018 to 2019. In comparison, prostate cancer screening increased by 10% from 2019 to 2020. While prostate cancer screening showed a continued positive increase during 2020, the relative increase was lower than in previous years.

Amongst people receiving screening for colorectal cancer, there was a SLR of 1.08. The screening gap was 1095.3 in females and 795.0 in males, while the SLRs were near-identical (Female: 1.09; Male: 1.07). The screening gap was 281.7 in Black patients, 863.3 in White patients, and 109.0 in Other patients, with similar SLRs (Black: 0.72; White: 0.86; Others: 0.92). In Hispanic patients, the screening gap and SLR were 47.3 and 0.67, respectively, compared to 1191.0 and 0.83 in Non-Hispanic patients.

The screening gap was negative, indicating absolute gains compared to the reference years, in nearly all subpopulations of men undergoing prostate cancer screening during the first four months of the pandemic in the US. Specifically, the screening gap and SLR were −21.0 and −0.22 in Black patients, −235.0 and −0.58 in White patients, −4.7 and −0.11 in Other patients, and −262.0 and −0.51 in Non-Hispanic patients, respectively. Patients with Hispanic ethnicity were the only group with screening losses, with a screening gap of 6.3 and SLR of 0.28.

**Figure 2:**
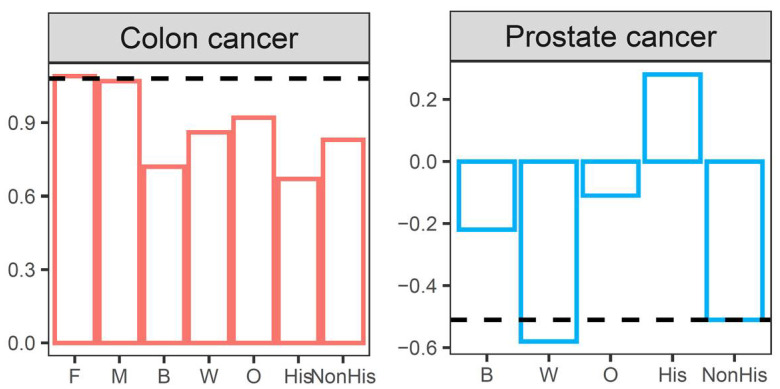
Cancer screening loss ratios for colon cancer and prostate cancer across sex, race, and ethnicity from March to June during 2020.

The screening loss ratios for colorectal cancer and prostate cancer across sex, race, and ethnicity are shown in Figure 2.

### 3.3. Cancer Screenings in 2020 Compared to Reference Years

**Figure 3:**
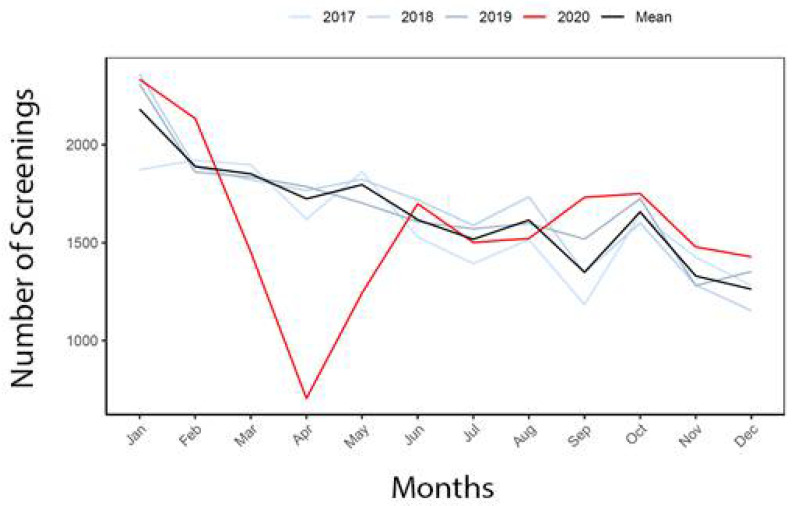
Colon cancer screening in 2020 compared to reference years.

Amongst patients undergoing screening for colorectal cancer, the 2020 yearlong screening gap was 815.0, which was improved from the acute, four-month gap of 1891.0 patients (Table 5). The screening gap was similar in males (404.7) and females (409.7), though the SLR was higher in males (Male: 0.58; Female: 0.43). The screening gap was 87.0 in Black patients, with a SLR of 0.23. The screening gap was negative, meaning higher than the reference years, in White patients (−327.7), Other patients (−66.0), Hispanic patients (−68.0), and Non-Hispanic patients (−242.7), with similar SLRs (White: −0.34; Others: −0.58; Hispanic −0.97; Non-Hispanic −0.18). Colon cancer screening loss and recovery over the year, compared to the reference years can also be seen in Figure 3.

**Figure 4:**
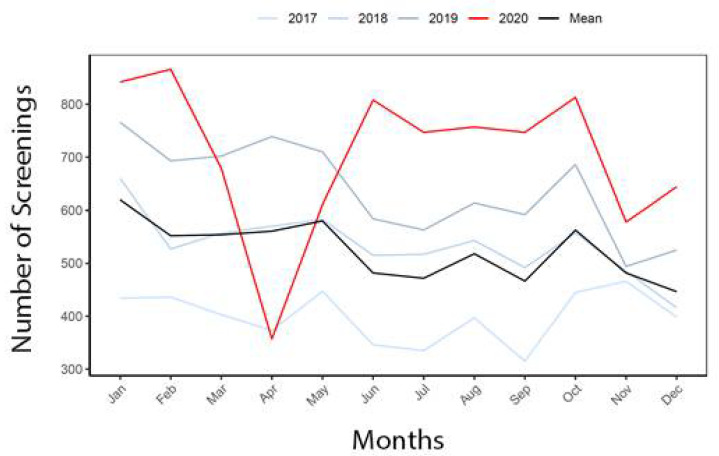
Prostate cancer screening in 2020 compared to reference years.

Screening increased for prostate cancer in 2020 compared to the reference years, with a yearlong screening gap of −2154.0 patients and yearlong SLR of −4.11 (Table 6). This is an increase compared to the screening gap of −278.7 patients during the four-month period at the start of the pandemic. The yearlong screening Gap for prostate cancer was −251.3 in Black patients, −1683.7 in White patients, and −161.7 in Other patients, but the SLR was higher in Black patients (Black: −2.82; White: −4.29; Others: −4.26). The yearlong screening gap for prostate cancer was −83.0 in Hispanic patients and −1972.3 in Non-Hispanic patients, with similar SLRs (Hispanic: −3.91; Non-Hispanic: −3.98). Prostate cancer screening loss and recovery over the year, compared to the reference years is shown in Figure 4.

**Figure 5:**
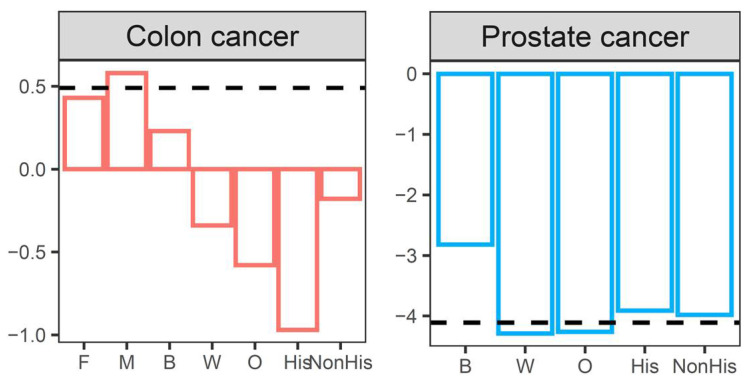
Cancer screening loss ratios for colon cancer and prostate cancer across sex, race, and ethnicity during 2020.

The screening loss ratios for colorectal cancer and prostate cancer across sex, race, and ethnicity for all of 2020 are depicted in Figure 5.

## 4. Discussion

### 4.1. Overview of Cancer Screening Delays

In this report we show the extent to which the COVID-19 pandemic impacted colorectal and prostate cancer screening in a large health system in the United States. During the pandemic, when healthcare workers and resources were diverted to infection control and resource conservation, there was a concomitant decrease in cancer screening. There were calls to mitigate against these diversions over concerns about delayed and missed diagnoses [6]. It was estimated that 37.7% of cancer operations globally (2,324,070 of 6,162,311) were delayed during the 12 week period of peak disruption and that it would take a median time of 45 weeks to resolve this backlog [7]. In this study, cancer screening activity returned to pre-pandemic rates approximately three to four months after the pandemic onset. By the end of 2020, the prostate cancer screening rate far exceeded the reference years by more than 2000 screenings. However, the rate of colorectal cancer screening did not increase enough to accelerate the catch-up, or alleviate pent-up demand for screenings that were missed during the first four months of the pandemic in the US.

### 4.2. Study Strengths

One of the unique aspects of this study was the method of calculating a SLR to determine the relative magnitude of change in screening. This contrasts with the many other studies describing cancer screening losses that focused on absolute screening deficits and percentage changes during the first peak of the pandemic and subsequent recovery periods [8,9,10,11]. One advantage of our methodology was in using the SLR tool to compare sex, race, and ethnicity subgroups within the study to identify and measure subtle screening disparities. These relative measures of screening loss could be compared regardless of the subgroup sizes and the ratios allow the reader to easily interpret the magnitude of the change in 2020 compared to the reference years within and across subgroups.

### 4.3. Health Disparities in Cancer Screening Delays and Recovery

With respect to specific populations, the data show that the pandemic was associated with a disproportionate decrease in colorectal cancer screening for Males and Black patients. For prostate cancer screening, there were gains in screening despite the pandemic. However, there was a lower year-over-year gain in 2020 compared to the years leading up to the pandemic, and Black men showed a disproportionately lower gain in prostate cancer screening.

Furthermore, evidence presented in this report shows that although screening for colorectal cancer returned to historical rates in June 2020, there was no increase in the rate in the latter part of 2020 to account for the acute losses immediately after the pandemic outbreak. The yearlong SLR of 0.49 for colorectal cancer screening suggests that screening for colorectal cancer needs to increase by approximately 50% to account for the screening deficit. These findings provide data that support some of the early pandemic predictions, and if extended, further raise concerns about excess deaths. In one such model, it was predicted that delays in cancer screening and treatment for breast and colorectal cancer would result in approximately 10,000 excess deaths (~1% increase in expected deaths from these cancers) [1]. Our findings were also consistent with other studies that demonstrated a substantial drop-off from March through June 2020, followed by a rapid increase in cancer screenings to rates similar to, or better than, those seen before the pandemic [3,4,5,8,9,10,11,12]. Other studies looking at the recovery period demonstrated a similar return to prior screening rates without a catch-up period to close the gap in missed screenings, particularly in colorectal cancer screening [8,9,12]. This widely recognized screening gap supports concerns about increased morbidity and mortality related to the delayed diagnosis of these cancers [13]. Ultimately, these data support enhancing investments in colorectal cancer screening efforts, which may take the form of stimulus funding and/or unifying efforts of various stakeholders such as payers, health systems, community-based organizations, and testing laboratories.

Delayed cancer screenings can be framed as a confluence of challenges to patients, health care professionals, and health systems, which may explain both the initial delays, as well as the incongruent recovery [14]. For instance, the impact of low socioeconomic resources such as loss of health insurance due to job loss, limited local healthcare resources including workforce and personal protective equipment (PPE), limited access to childcare or senior care limit scheduling or rescheduling screening in certain groups [14]. During the pandemic, people were also reluctant to attend in-person office visits due to fears of COVID-19 transmission, further contributing to delays in routine screening. There has also been a reduction in healthcare workforce numbers during the pandemic due to COVID-19 illness, quarantining, furloughs, early retirements, career changes, and hiring freezes [14]. Furthermore, social distancing practices and limited PPE may have restricted the ability of health centers to catch up, even after legal limitations to elective procedures were lifted.

The reasons for these delays also, presumably, differed between the cancer groups. Delays in colorectal cancer screening with subsequent delayed catch-up may have been impacted by the long time-intervals between tests, invasive nature of colonoscopy screening, and ongoing intermittent bans and delays in elective procedures during 2020. In contrast, the less-invasive prostate cancer screenings by PSA testing may have rebounded quickly due to increases in telemedicine utilization or other test-specific factors.

Just as the reasons for cancer screening delays vary by population, cancer type, and testing modality, the efforts to accelerate the recovery from these delays will require a similarly diversified approach [15]. The first step will be ensuring safe office practices to prevent transmission of COVID-19 and clear communication of these measures to patients. These efforts will likely involve personal protective equipment, limiting patient time in waiting rooms, and single-problem visits targeted at cancer screening. Communication from the top of the health system to all providers must be clear. Next, health systems will need to prioritize diagnostic testing for potentially missed or progressed disease from delayed cancer screenings. Finally, there must be targeted outreach to high-risk patients and those most overdue for screening, with an emphasis on educating and screening in underserved communities. These and other measures to close the gap in cancer screening have been outlined by groups within the National Cancer Institute [15]. There may also need to be an investment of financial resources to expand cancer screening through federal funding.

### 4.4. Study Limitations

While we took efforts to minimize bias, there were several limitations to this study design. This was a single-center study with patients largely localized in a single state within the United States. The retrospective database study design meant that individual patient-level data was not manually reviewed. Any type of screening assessment was accepted, regardless of the screening frequency standards, meaning that changing practice patterns (e.g., changing the usage of CT colonography, which is done every five years while a colonoscopy is done every ten years) and disparate indications for screening (e.g., baseline risk factors or early follow-up for repeat screening) may have impacted our findings. A key assumption was the use of the first diagnosis or procedural code within a calendar year for screening and this may explain the skew of screening tests earlier in the year. This also meant that repeat screenings within one calendar year could not be identified or counted in the study data.

## 5. Conclusions

In sum, colorectal cancer screening rapidly decreased but recovered quickly and prostate cancer screening went up during the first US peak of the COVID-19 pandemic and continued to increase throughout 2020 across all groups. Cancer screening in males and black patients was disproportionately affected by the pandemic. Interventions are needed urgently to stimulate cancer screening to above historical reference rates to account for the deficits incurred immediately after the COVID-19 outbreak and to avoid excess, preventable deaths.

## Figures and Tables

**Table 1:** Colorectal cancer screening encounters from 2017 to 2019.

Variable	Category	Count	Percentage
Sex	FEMALE	34,019	57.3
	MALE	25,355	42.7
	Missing	5	0
Race	BLACK	13,449	22.6
	OTHER	4110	6.9
	WHITE	34,900	58.8
	Missing	6920	11.7
Ethnicity	HISPANIC	2511	4.2
	NOT HISPANIC	49,732	83.8
	Missing	7136	12

**Table 2:** Prostate cancer screening encounters from 2017 to 2019.

Variable	Category	Count	Percentage
Race	BLACK	3212	17
	OTHER	1366	7.2
	WHITE	14,125	74.8
	Missing	182	1
Ethnicity	HISPANIC	765	4.1
	NOT HISPANIC	17,861	94.6
	Missing	259	1.4

**Table 3:** Number of colorectal cancer screenings from March to June during 2020.

	Reference (Yearly Average) ^1^	2020 ^2^	Reference (Monthly Average) ^3^	Gap ^4^	Screening Loss Ratio ^5^
All ^6^	6988.0	5097	1747.0	1891.0	1.08
Female	4006.3	2911	1001.6	1095.3	1.09
Male	2981.0	2186	745.2	795.0	1.07
Black	1574.7	1293	393.7	281.7	0.72
White	4020.3	3157	1005.1	863.3	0.86
Others	473.0	364	118.2	109.0	0.92
Hispanic	282.3	235	70.6	47.3	0.67
Non-Hispanic	5759.0	4568	1439.8	1191.0	0.83

^1^ “Reference (yearly average)” is the yearly average number of screenings among reference years (2017 to 2019) focusing on March to June only. ^2^ “2020” is total number of screenings in 2020 focusing on March to June only. ^3^ “Reference (monthly average)” is calculated through dividing “Reference (yearly aver-age)” by 4 (March, April, May, and June) to obtain monthly average screening number among reference years. ^4^ “Gap” is calculated through subtracting “2020” from “Reference (yearly average)” to represent total number of losses in March to June, 2020. Positive numbers represent screening losses and negative numbers represent screening gains. ^5^ “Screening Loss Ratio” is calculated through dividing “Gap” by “Reference (monthly average)”. Positive numbers represent screening losses and negative numbers represent screening gains. ^6^ Sex missingness: 2 (0%) in reference years, 0 (0%) in 2020; Race missingness: 2760 (13.2%) in reference years, 283 (5.6%) in 2020; Ethnicity missingness: 2840 (13.5%) in reference years, 294 (5.8%) in 2020.

**Table 4:** Number of prostate cancer screenings from March to June during 2020.

	Reference (Yearly Average) ^1^	2020 ^2^	Reference (Monthly Average) ^3^	Gap ^4^	Screening Loss Ratio ^5^
All ^6^	2176.3	2455	544.1	−278.7	−0.51
Black	381.0	402	95.2	−21.0	−0.22
White	1615.0	1850	403.8	−235.0	−0.58
Others	164.3	169	41.1	−4.7	−0.11
Hispanic	91.3	85	22.8	6.3	0.28
Non-Hispanic	2058.0	2320	514.5	−262.0	−0.51

^1^ “Reference (yearly average)” is the yearly average number of screenings among reference years (2017 to 2019) focusing on March to June only. ^2^ “2020” is total number of screenings in 2020 focusing on March to June only. ^3^ “Reference (monthly average)” is calculated through dividing “Reference (yearly average)” by 4 (March, April, May, and June) to obtain monthly average screening number among reference years. ^4^ “Gap” is calculated through subtracting “2020” from “Reference (yearly average)” to represent total number of losses in March to June, 2020. Positive numbers represent screening losses and negative numbers represent screening gains. ^5^ “Screening Loss Ratio” is calculated through dividing “Gap” by “Reference (monthly average)”. Positive numbers represent screening losses and negative numbers represent screening gains. ^6^ Race missingness: 48 (0.7%) in reference years, 34 (1.4%) in 2020; Ethnicity missingness: 81 (1.2%) in reference years, 50 (2%) in 2020.

**Table 5:** Number of colon cancer screenings during the 2020 calendar year.

	Reference (Yearly Average) ^1^	2020 ^2^	Reference (Monthly Average) ^3^	Gap ^4^	Screening Loss Ratio ^5^
All ^6^	19,793.0	18,978	1649.4	815.0	0.49
Female	11,339.7	10,935	945.0	404.7	0.43
Male	8451.7	8042	704.3	409.7	0.58
Black	4483.0	4396	373.6	87.0	0.23
White	11,633.3	11,961	969.4	−327.7	−0.34
Others	1370.0	1436	114.2	−66.0	−0.58
Hispanic	837.0	905	69.8	−68.0	−0.97
Non-Hispanic	16,577.3	16,820	1381.4	−242.7	−0.18

^1^ “Reference (yearly average)” is the yearly average number of screenings among reference years (2017 to 2019). ^2^ “2020” is total number of screenings for all of the year 2020. ^3^ “Reference (monthly average)” is calculated through dividing “Reference (yearly average)” by 12 months to obtain monthly average screening number among reference years. ^4^ “Gap” is calculated through subtracting “2020” from “Reference (yearly average)” to represent total number of losses in 2020. Positive numbers represent screening losses and negative numbers represent screening gains. ^5^ “Screening Loss Ratio” is calculated through dividing “Gap” by “Reference (monthly average)”. Positive numbers represent screening losses and negative numbers represent screening gains. ^6^ Sex missingness: 5 (0%) in reference years, 1 (0%) in 2020; Race missingness: 6920 (11.7%) in reference years, 1185 (6.2%) in 2020; Ethnicity missingness: 7136 (12%) in reference years, 1253 (6.6%) in 2020.

**Table 6:** Number of prostate cancer screenings during 2020.

	Reference (Yearly Average) ^1^	2020 ^2^	Reference (Monthly Average) ^3^	Gap ^4^	Screening Loss Ratio ^5^
All ^6^	6295.0	8449	524.6	−2154.0	−4.11
Black	1070.7	1322	89.2	−251.3	−2.82
White	4708.3	6392	392.4	−1683.7	−4.29
Others	455.3	617	37.9	−161.7	−4.26
Hispanic	255.0	338	21.2	−83.0	−3.91
Non-Hispanic	5953.7	7926	496.1	−1972.3	−3.98

^1^ “Reference (yearly average)” is the yearly average number of screenings among reference years (2017 to 2019). ^2^ “2020” is total number of screenings for all of the year 2020. ^3^ “Reference (monthly average)” is calculated through dividing “Reference (yearly average)” by 12 months to obtain monthly average screening number among reference years. ^4^ “Gap” is calculated through subtracting “2020” from “Reference (yearly average)” to represent total number of losses in 2020. Positive numbers represent screening losses and negative numbers represent screening gains. ^5^ “Screening Loss Ratio” is calculated through dividing “Gap” by “Reference (monthly average)”. Positive numbers represent screening losses and negative numbers represent screening gains. ^6^ Race missingness: 182 (1%) in reference years, 118 (1.4%) in 2020; Ethnicity missingness: 259 (1.4%) in reference years, 185 (2.2%) in 2020.

## Data Availability

Not applicable.

## Supplementary Materials

The following supporting information can be downloaded at: https://www.mdpi.com/article/10.3390/healthcare10020264/s1, Table S1: Number of Cancer Screening Encounters by Year.
