# Supplementary material for: Impact of the COVID-19 Pandemic on Colorectal and Prostate Cancer Screening in a Large U.S. Health System
Source: Healthcare (Basel). 2022 Jan 29;10(2):264. doi: 10.3390/healthcare10020264 (PMC8871935; doi:10.3390/healthcare10020264)
Supplement: Supplementary file 1 [file healthcare-10-00264-s001.zip › healthcare-1539467-supplementary.pdf]

Supplementary

Codes included current procedural terminology (CPT) codes, healthcare common procedure coding system (HCPCS), and International Classification of Diseases 10 (ICD-10) codes for colon cancer screening (CPT Code 45378, HCPCS Code G0105, HCPCS Code G0121, CPT Code 82270 (gFOBT), CPT Code 82274 (FIT), HCPCS Code G0328, ICD-10 code Z12.11), breast cancer screening (CPT Code 77067, CPT Code 77063, HCPCS Code G0202, HCPCS Modifier GH, HCPCS Modifier GG, HCPCS Code G9899, HCPCS Code G9900, ICD-10 Code Z12.31, ICD-10 Code Z12.39, HCPCS Code G0101), cervical cancer screening (HCPCS Code P3001, HCPCS Code Q0091, HCPCS Code P3000, HCPCS Code G0476, G0123, G0143, G0144, G0145, G0147, G0148, G0124, G0141, Z01.411, Z01.419, Z11.51, Z12.4, HCPCS Code G0101), prostate cancer screening (HCPCS code G0102 (DRE), HCPCS code G0103 (PSA), ICD-10 code Z12.5), and lung cancer screening: HCPCS code G0296, HCPCS code G0297 (LDCT)).

For the purposes of comparing demographics to our data set, we utilized the most recent United States Census Bureau estimates for Alachua County (https://data.census.gov/cedsci/profile?g=0500000US12001). This survey reports an estimated population of 265,443 in Alachua County, out of an estimated population of 2,293,391 in the 23 counties. Of that population, race distribution is estimated to be 68.9% ± 0.3% White, 20.1% ± 0.2% Black, 0.4% ± 0.1% American Indian, 6.1% ± 0.2% Asian, 0.1% ± 0.1% Native Hawaiian and Other Pacific Islander, 1.1% ± 0.2% some other race, and 3.4% ± 0.3% two or more races. Of that population, 9.9% are estimated to be of Hispanic ethnicity. Of that population, 8.4% are estimated to not have health insurance, though this likely changed significantly during the pandemic.

Supplemental Table S1. Number of Cancer Screening Encounters by Year.

	**2015**	**2016**	**2017**	**2018**	**2019**	**2020**
**Colon**	6771	13369	19147	20084	20148	18978
**Prostate**	2445	4260	4795	6422	7668	8449
